# Comparison of Immune‐Related Responses of Several Slow‐Growing Indigenous Chickens With a Fast‐Growing Broiler in Iran

**DOI:** 10.1002/vms3.70288

**Published:** 2025-03-03

**Authors:** Donya Khazali, Alireza Ehsani, Ali Akbar Masoudi, Mohammad Amir Karimi Torshizi, Rasoul Vaez Torshizi, Peymaneh Davoodi, Alireza Valizadeh, Seyed Mohammad Ali Sheykh‐Al‐Eslami

**Affiliations:** ^1^ Department of Animal Science Tarbiat Modares University Tehran Iran

**Keywords:** cellular immunity, fast‐growing broiler, humoral immunity, immunoglobulin, slow‐growing

## Abstract

Relationship between immunity and growth rate in chickens is complex and multifaceted. Chicken's immune system plays a crucial role in its overall health, well‐being and ability to grow and develop properly. On the other hand, indigenous chickens (ICs) are slow growing but considered to have higher immunocompetence compared to fast‐growing commercial broilers. Therefore, this study aimed to evaluate immune responses to sheep red blood cell (SRBC) and phytohaemagglutinin (PHA) in slow‐growing chickens, including five different Iranian ecotype ICs and a fast‐growing broiler of Ross 308 (R). A total of 600 chicks, 100 from each sex and strain, were raised under identical environmental and management conditions after hatching eggs from five distinct Iranian ICs from West Azerbaijan (W), Khorasan (K), Fars (F), Isfahan (I), Mazandaran (M) and a commercial Ross 308 were collected. To investigate humoral immune responses, two SRBC injections were administered at ages 53 and 60 days, and at 67 days, the haemagglutination method was used to assess immunoglobulin Y (IgY), immunoglobulin M (IgM) and total immunoglobulin (IgT). Additionally, a commercial kit and microplate reader were used to assess the amounts of total protein and albumin/globulin. Cellular immune responses were evaluated by administering a PHA injection. Mainly SRBC responses and plasma protein levels in ICs were higher than R. The PHA responses of R and W were higher than other ICs (*p* < 0.05). These significant differences may confirm that the immunocompetence of ICs are higher than that in the commercial lines.

## Graphical abstract

This study focuses on the complex relationship between immunity and growth rate in chickens, particularly comparing five Iranian indigenous ecotypes with the fast‐growing Ross 308 broilers. It evaluates immune responses to sheep red blood cells and phytohaemagglutinin, finding that indigenous chickens exhibit superior humoral immune responses. The results suggest that these indigenous strains of Iran possess higher immunocompetence in comparison to the commercial strain.

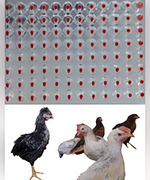

## 1 Introduction

Slow‐growing chickens have been becoming of more interest compared to fast‐growing commercial breeds because of their flavoursome meat, higher nutritional value, firmer texture and welfare (Kaewsatuan et al. 2022). Modern broiler chickens can already achieve market weight (>2 kg) in as little as 6 weeks due to genetic selection for higher growth rate, improving feed quality and management. These increases in production and efficiency, however, come at a cost to broiler health and welfare when compared to broiler chickens from the 1950s. It also comes with some welfare concerns and they seem to have a weaker immune system and greater mortality rates (Dawson et al. 2021), especially in outdoor semi‐extensive and free‐range production systems (Hofmann et al. 2020).

The immune system response could be divided into innate and adaptive responses. The innate responses are older in the evolution history and prime to act against pathogen through enzymes and phagocytosis strategy in the circulatory system (Heaton et al. 2016). The adaptive immune response includes cellular and humoral immunity. Although cellular immunity using the phagocytosis mechanism destroys the cell that contains pathogenic particles, the cell‐mediated immune response is a basic and nonspecific body response to microbial infection with the help of lymphocytes including T cells (Pathak et al. 2018). This is a key function to resist the organisms against external antigens (Raeesi et al. 2019). From various innate immune tests, the measurements of immunoglobulin Y (IgY) and immunoglobulin M (IgM) and the response to sheep red blood cell challenge (SRBC) are widely applicable (Sahin and Ozturk 2018). SRBC challenge is widely used to assess the humoral response without causing illness (Li et al. 2000; Radhika et al. 2017; Raeesi et al. 2019).

One useful method for evaluating the animal health, the degree of damage to various organs and the progress of disease, is blood biochemistry analysis. Actually, the blood concentration of biochemical molecules is usually constant, but factors, such as breed, age, sex, season, nutritional status and climate, change this concentration (Kashap et al. 2017). Studies showed that different species and breeds may react differently to pathogens because of their genetic architecture and their evolution process (Divya et al. 2018). It means that genetic selection may improve immune responses to pathogens (Rauw 2012; de Klerk et al. 2018; Raeesi et al. 2019).

Indigenous chickens (ICs) are one of the main sources of animal protein in developing countries. The importance of using animal breeding programmes for increasing the genetic potential of ICs is popular in most countries (Vali 2008). IC farming in rural areas has adapted to birds for several years in different regions (Sørensen 2010; Morammazi and Habibi 2017). The genetic sources of Iranian ICs have been reported to have a long history with a high range of diversity (Shahbazi et al. 2007).

In Iran, there are various IC ecotypes. The comparative analysis of immune responses between IC ecotypes and commercial strains, particularly the Ross 308 broiler, reveals significant insights into their health dynamics. Our study uniquely examines five distinct Iranian IC ecotypes, highlighting their superior immunocompetence despite slower growth rates (Sadr et al. 2023). ICs are known for their resilience to diseases and ability to thrive in harsh conditions (Mengesha 2012; Suganthi 2014; Morammazi and Habibi 2017). By measuring IgY and IgM, we provide a nuanced understanding of how these strains respond to immune challenges (Vaillant et al. 2022; Hinkle and Cheever 2014). Our findings fill a gap in the literature regarding immunity and growth, offering practical implications for enhancing poultry health through targeted breeding programmes (Fitzgerald et al. 2019; Loh et al. 2013). This research contributes valuable insights for sustainable poultry production in regions where indigenous breeds are integral to local agriculture.

## 2 Materials and Methods

### 2.1 Birds Husbandry

This experimental research was carried out in the animal science department of relevant university, from June to September. Eggs from five ICs of West Azerbaijan (W), Mazandaran (M), Khorasan (K), Isfahan (I) and Fars (F) and the commercial strain of Ross 308 (R) were collected from breeding stations with mothers at the same age, and 100 chicks from each were raised under the same condition until the end of the experiment. At the age of 53 days old, 36 birds from each group were randomly selected for immunity response experiments.

As native chickens belong to five groups from different regions of the country, rearing condition was managed to resemble rural management for such chickens in Iran. The Ross 308 strain was also reared at the same condition to have a uniform condition for all chickens under this study. Chickens were raised indoor in six separates floor pens that contained watering nipples, manual feeding and electric fans for ventilation and cooling. The raising density of each pen was 8 birds/m^2^, under the average temperature of 28°C at the time of challenges, and the photoperiod was 23 h at the beginning for 1‐day‐old chicks and reduced to 14 h at the time of challenging period. The current investigation employed a uniform environment to assess the immune response of native chickens and commercial Ross chickens.

All birds were fed with the same starter, grower and finisher ad libitum (Table 1) to ensure consistent feeding conditions, as native chickens do not have established feed requirements. The post‐hatch starter was changed to grower at Week 3, and the finisher diet was introduced at Week 7, and continued thereafter. Antibiotics were not administered during the experiment, and water was freely accessible to the birds.

**TABLE 1:** Dietary components for indigenous and commercial chickens.

Compounds (kg/t)	Starter	Grower	Finisher
Corn	553	580.5	592
Soybean	375	340	310
Bran	10	10	20
Oil	20	30	40
Calcium phosphate	18	17	16
Calcium carbonate	5	5	5
Methionine	4	3	2.5
Lysine	2	1.5	1.5
Threonine	1	1	1
Mineral and vitamin supplements	5	5	5
Multi enzyme	0.5	0.5	0.5
Anti‐coccidiosis	0.5	0.5	0.5
NaCl	4	4	4
Sodium bicarbonate	2	2	2
%CP	21.5	19.5	18
En (kcal)	2800	2950	3000

### 2.2 SRBC Challenge

For the SRBC suspension preparation, blood was obtained from healthy ram maintained at the university farm. About 30 mL blood in anticoagulant EDTA (Ethylenediaminetetraacetic acid) solution was collected from the jugular vein. The red blood cells were washed three times with phosphate buffer saline (PBS). After the final wash, the concentration of red cells was brought to 5% in PBS. At 53 and 60 days of their age, chicks were injected with 0.2 mL of SRBC of 5% suspension on the right side of the breast muscle. Seven days after the second injection, 2 mL blood samples were collected from the wing vein of the chickens in microtubes containing EDTA as an anticoagulant.

The blood samples were centrifuged at 6000 rpm for 5 min to harvest plasma. Plasma harvesting stored at 4°C and incubated at 56°C for 30 min, in order to inactive complement system using haemagglutination inhibition (HI) method (Wegmann and Smithies 1966). According to this method, the total antibody level was determined by the agglutination of plasma samples using PBS and SRBC 1%. A total of 2‐mercaptoethanol was used instead of PBS to detect susceptible antibody M and resistant antibody Y.

### 2.3 Plasma Proteins

Birds’ plasma samples at age of 67 days from 208 birds were used to measure concentration of total protein and albumin by using Pars Azmoon commercial kits. Bromocresol green colorimetric method was determined by the albumin assay kit. In this method, the amount of 10 µL of the plasma sample was added to 1 mL of bromocresol green colouring solution. The samples were then stored at 37°C for 10 min. Finally, the absorbance intensity of the samples was read by the microplate reader at a wavelength of 545 nm.

The intensity of the created colour is proportional to the amount of albumin in the sample. Total protein concentration was measured using the Total protein kits. The protein in the alkaline environment with copper ions paints an azure complex. The intensity of the colour created is proportional to the protein content in the sample. The amount of 20 µL of plasma was incubated with 1 mL mixture solution and 5 min stored at 37°C. Standard optical absorption of samples was measured against Blanc with microplate reader. Globulin concentration was determined by reducing albumin concentration from total protein. Also, albumin‐to‐globulin ratio was calculated.

### 2.4 Phytohaemagglutinin (PHA) Challenge

To measure cellular immune responses, PHA is widely used. We used PHA and PBS injection to all bird toe web between the second and third digits of both left and right feet at the age of 67 days. A volume of 0.1 mL PBS was injected to the left foot toe web as the control foot, and 0.1 mL PHA was injected to the right foot as the test foot. The thickness of both feet toes web was measured before the injection and 24 h after injection by using a digital caliper. Toe index was calculated as

$$\mathrm{TI}=\left(\mathrm{RAP}-\mathrm{RBP}\right)-\left(\mathrm{LAS}-\mathrm{LBS}\right)$$

Where TI stands for toe index, the index of toe web thickness or PHA stimulation index in millimetres, RAP is the thickness of the right foot toe web after injection of PHA, RBP is the thickness of the right foot toe web before the injection of PHA, LAS is the thickness of left foot toe web after injection of PBS, and LBS is the thickness of left foot toe web before injection of PBS.

### 2.5 Statistical Analysis

Statistical analyses of all data were performed using R lsmeans and multcomp packages and assessed with a general linear model (GLM). All values were expressed as the least‐squares means ± SE. Differences between the means of groups were considered by Tukey's multiple range test using agricolae package and were significant at *p < 0.05:

$$Y_{ijk}=\mu +S_{i}+G_{j}+SG_{ij}+b{\left(W\right)}_{k}+e_{ijk}$$

Where *Y_ijk_* is the immune response measurements, *μ* overall mean, *S_i_* is the effect of breed, *G_j_* is the effect of gender, *SG_ij_* is the interaction of gender and breed, *b*(*W*)*_k_* is the weight effect as a covariate and *e_ijk_* is the residual error. Given that the R commercial chicken exhibits a significantly greater weight compared to the native breeds, the body weight at 49 days of age is employed as a covariate in the analysis, as presented in Table 2.

**TABLE 2:** Commercial and indigenous chicken weights at 49 days of age in grams.

Strain	Mean	Standard deviation	Maximum	Minimum
Ross 308	1934.74	220.46	2380	1500
Fars	593.88	86.54	800	460
Isfahan	477.4	86.56	680	344
Khorasan	576.11	91.62	800	420
Mazandaran	616.67	102.32	903	460
West Azerbaijan	703.71	105.41	925	535

## 3 Results and Discussion

### 3.1 Humoral Response to SRBC

For the reason that the interaction between gender and breed was not significant (*p* > 0.05); therefore, the interaction effect was removed from the analysis. The range of HA in the present study was less than the average HA titre reported by Becker et al. (2011) (12.38 ± 0.60) in the Indian Aseel breed. As their studied birds were more than 300 days old, they claimed that older chicks have higher antibody responses compared to younger chicks. The reason may be due to the development of memory cells and exposure to varied antigenic determination in older chickens (Becker et al. 2011).

Antibodies produced in the secondary response to SRBC at the age of 67 days are shown in Table 3. Total antibodies and IgY titres showed a significant difference between the studied populations. The total antibody of W and M chicks were the highest and had a significant difference with R (*p *< 0.05). Moreover, the IgY titre was the highest in W, F and M populations showing a significant difference with the R birds (*p* > 0.05). According to previous studies, the immune response and antibody production in birds can be influenced by various factors such as breed, stressors, high stocking density, diseases and hosing environment (Leandro et al. 2011; Hofmann et al. 2020). In another study by Emam et al. (2013), the humoral immunity of IgY in W, I, R and Cobb commercial strains, the response intensity in the first two indigenous populations were significantly higher than commercial strains (Emam et al. 2013).

**TABLE 3:** Least‐squares means ± SE of humoral response to sheep red blood cell (SRBC) in log_2_ of native chickens and Ross commercial strain.

Fixed effects	IgT	IgY	IgM
Breed			
Ross	5.02^b^ ± 1.34	2.95^b^ ± 1.06	2.06 ± 0.86
Isfahan	6.13^ab^ ± 0.49	3.80^ab^ ± 0.39	2.33 ± 0.33
Fars	6.55^ab^ ± 0.39	4.18^a^ ± 0.31	2.37 ± 0.25
Khorasan	6.46^ab^ ± 0.40	3.73^ab^ ± 0.31	2.72 ± 0.26
Mazandaran	6.63^a^ ± 0.39	4.24^a^ ± 0.30	2.39 ± 0.25
West Azerbaijan	7.13^a^ ± 0.31	4.52^a^ ± 0.25	2.61 ± 0.20
Sex			
Male	6.42 ± 0.17	4.01 ± 0.13	2.40 ± 0.11
Female	6.22 ± 0.18	3.79 ± 0.14	2.42 ± 0.12

*Note*: a, b, ab letters show significant differences between averages. averages that have one letter in common are not significantly different. The averages are significantly different if they have no letter in common.

Abbreviations: IgM, immunoglobulin M; IgT, total immunoglobulin; IgY, immunoglobulin Y.

There was no significant difference in IgM titre among the groups in this study. The reason for that might be due to the fact that IgM is the first globulin that confronts the antigen to generate other or secondary antibodies accordingly. It can be expected, there is always a basic level of IgM in the plasma (Davison et al. 2008; Cruse and Lewis 2010) which makes the differences insignificant.

As another finding, males have revealed a higher total immunoglobulin (IgT) and IgY, in contrast to the IgM in comparison with females, although the sex‐specific difference was not statistically significant in this study. The challenged birds were at 53–67 days of age that sexual maturity is not reached; therefore, they did not show sex differences significantly. The effect of sex on the immune response to SRBC was reported by previous studies (Gross et al. 1980; Van der Zijpp and Leenstra 1980; Pinard et al. 1992; Divya et al. 2018). All of five indigenous ecotypes in this study were colourful versus white colour of R. Reports showed that the immunity responses and feather pigmentation are interconnected; therefore, it can be a source of higher immunity responses in the indigenous ecotypes in this study (Davoodi et al. 2022).

### 3.2 Blood Protein Concentration

Blood protein levels in chickens are important indicators of health status and can represent a basis identification of metabolic variations (Tóthová et al. 2019). In human biology, higher globulin levels and lower A/G ratio were reported to be strongly associated with the risk of some diseases (Ye et al. 2020). We assessed the protein profile in ICs and R chickens to scan their health status after immune challenges. Total protein, albumin and globulin levels in 1 g per 100 mL plasma, and the ratio of albumin to globulin at 67 days of age are shown in Table 4. Total protein concentration in the I population was significantly higher than R, K and F (*p *< 0.05). Males had a lower total protein concentration than females, but this difference was not significant. Albumin concentration in the native population of I was higher than all populations, and there was a significant difference with the R and K and M (*p *< 0.05).

**TABLE 4:** Least‐squares means ± SE of plasma proteins in gram in 100 µL of native chickens and Ross commercial strain.

Fixed effects	Total protein	Albumin	Globulin	Albumin/Globulin
Breed				
Ross	3.76^b^ ± 0.28	3.10^d^ ± 0.20	0.66^b^ ± 0.30	4.23 ± 1.64
Isfahan	5.38^a^ ± 0.10	3.71^a^ ± 0.07	1.67^a^ ± 0.11	2.86 ± 0.61
Fars	4.94^b^ ± 0.08	3.57^abc^ ± 0.06	1.40^ab^ ± 0.08	3.01 ± 0.48
Khorasan	5.04^ab^ ± 0.08	3.41^cd^ ± 0.06	1.62^ab^ ± 0.09	2.27 ± 0.49
Mazandaran	5.00^ab^ ± 0.08	3.50^bc^ ± 0.06	1.50^ab^ ± 0.08	2.35 ± 0.47
West Azerbaijan	5.02^ab^ ± 0.06	3.59^ab^ ± 0.04	1.42^ab^ ± 0.07	2.71 ± 0.38
Sex				
Male	4.78 ± 0.03	3.46 ± 0.02	1.32 ± 0.03	3.16 ± 0.21
Female	4.95 ± 0.03	3.50 ± 0.02	1.44 ± 0.04	2.65 ± 0.22

*Note*: a, b, c, d, ab, bc, abc, and cd, letters show significant differences between averages. averages that have one letter in common are not significantly different. The averages are significantly different if they have no letter in common.

Albumin concentration was significantly higher in of W than K and R. Furthermore, the amount of albumin concentration in F and M was significantly higher than the R. Although the albumin concentration in females was higher than that of males, the difference was not significant. The concentration of globulin was significantly different among the studied populations, the lowest concentration level was in the commercial strain of R and the highest amount was appeared in I. The concentration of globulin in females was lower than that of males, but not significant.

There was no significant difference of the albumin to globulin ratio among the populations and also in different sexes. Blood proteins in birds are a crucial biochemical component that can be used to identify metabolic changes and serve as an important health status indicator (Tóthová et al. 2019). Animals’ plasma immunoglobulin concentrations can be used as a measure to determine their humoral immunological state (Gong et al. 2018). This result was also obtained in the present study and the humoral immune response and the concentration of plasma proteins were higher in native chickens than in Ras strain. Strain had effect on total protein, and albumin. This was also reported by Olaniyi et al. (2012). The typical concentration of blood albumin in ICs in this study is similar level that reported by Ismoyowati et al. (2022).

Serum protein consists primarily of albumin and globulin, which play important roles in nutrition and immune function. A decrease in albumin level leads to the development of hypoproteinemia. Also, an elevation in blood albumin level suggests the existence of an infection inside the animal's body. The ratio of albumin to globulin has been identified as a prognostic indicator in different types of cancer in human (He et al. 2017).

### 3.3 Cellular Response to PHA

The intensifying of the increased thickness of the toe web indicates a strong cellular immune response in the chicken (Martin et al. 2006). The least‐squares of the toe web thickness index as a response to the cellular immune and plasma protein concentration of all birds at 67 days of age are shown in Table 5. That shows a significant difference among studied populations. R and W populations have revealed the highest thickness and significant difference with M, K and I populations. Also, this index was significantly lower in K than in other populations (*p *< 0.05). There was no significant sex‐specific difference in cellular immune response.

**TABLE 5:** Least‐squares means ± SE of cellular response to phytohaemagglutinin (PHA) in mm of native chickens and Ross commercial strain.

Fixed effects	FI
Breed	
Ross	0.97^a^ ± 0.23
Isfahan	0.63^b^ ± 0.08
Fars	0.72^ab^ ± 0.07
Khorasan	0.32^c^ ± 0.07
Mazandaran	0.57^b^ ± 0.06
West Azerbaijan	0.86^a^ ± 0.05
Sex	
Male	0.68 ± 0.03
Female	0.68 ± 0.03

*Note*: a, b, c, and ab letters show significant differences between averages. averages that have one letter in common are not significantly different. The averages are significantly different if they have no letter in common.

Emam et al. (2013) reported that the cellular immune response was measured by injecting PHA into the toe web in the native populations of W, I and M with commercial strains of R and Cobb. They reported that after 48 h of injection, the severity of response in commercial strains and the indigenous population of W were significantly higher than the M and I populations. Our results were in agreement with their findings (Emam et al. 2013).

The researchers found that the selection of chickens for higher production can cause a negative effect on the humoral immune response to SRBC antibodies, while it had no significant effect on cellular immune responses (Qureshi and Havenstein 1994; Cheema et al. 2003; Mohammadi‐Tighsiah et al. 2018). In another study by Yadav et al. (2018), it is reported that cell‐mediated immune response to PHA is positively associated with body weight (Yadav et al. 2018).

## 4 Conclusions

In the present study, a comparison of two humoral and cellular immune responses were performed among five Iranian native chickens and Ross commercial strains. Collectively, native chicken populations showed a higher immune performance compared to the commercial R 308 strain. In more detail, native chickens had the higher humoral immune responses than R population, whereas cellular immune response in some ICs revealed similar pattern as R. In total, our findings shed some light in a way of discovery of natural potential of Iranian native chickens and getting advantages of the higher immunity potential of them to survive in extreme conditions, which will enable us to perform breeding schemes for generating novel crossbred chickens that have both high productivity and immunocompetence to reach the sustainable poultry industry.

## Author Contributions

**Donya Khazali**: conceptualization, data curation, formal analysis, investigation, methodology, resources, software selection, validation, visualization, writing – original draft, writing the manuscript. **Alireza Ehsani**: conceptualization, data curation, formal analysis, funding acquisition, investigation, methodology, project administration, resources, software, supervision, validation, visualization, writing – original draft, writing – review and editing the manuscript. **Ali Akbar Masoudi**: conceptualization, data curation, formal analysis, methodology, review and editing the manuscript. **Mohammad Amir Karimi Torshizi**: conceptualization, data curation, methodology, software, writing – review and editing. **Rasoul Vaez Torshizi**: data curation, formal analysis, software. **Peymaneh Davoodi**: data curation, methodology, writing – review and editing. **Alireza Valizadeh**: data curation, funding acquisition, methodology, resources. **Seyed Mohammad Ali Sheykh‐Al‐Eslami**: conceptualization, data curation, formal analysis, funding acquisition, methodology, resources, software.

## Ethics Statement

Hereby, I, Alireza Ehsani, consciously assure that for the manuscript ‘Comparison of immune‐related responses of several slow‐growing indigenous chickens with a fast‐growing broiler in Iran’ the following are fulfilled:

1. This material is the authors’ own original work, which has not been previously published elsewhere.
2. The article is not currently being considered for publication elsewhere.
3. The article reflects the authors’ own research and analysis in a truthful and complete manner.
4. The article properly credits the meaningful contributions of co‐authors and co‐researchers.
5. The results are appropriately placed in the context of prior and existing research.
6. All sources used are properly disclosed (correct citation). Literally copying of text must be indicated as such by using quotation marks and giving proper reference.
7. All authors have been personally and actively involved in substantial work leading to the article, and will take public responsibility for its content.
8. The Ethics Committee on the Use of Animals of Tarbiat Modares University, Iran approved all the experiments and protocols used in this study with approval number 9530371003.

## Conflicts of Interest

The authors declare no conflicts of interest.

### Peer Review

The peer review history for this article is available at https://publons.com/publon/10.1002/vms3.70288.

## Sanctions Law and Regulations

The authors are employed in ‘Tarbiat Modares University’ a public institution that research or education is the onlyfunction of the entity and have no connection with governmental organizations.

## Data Availability

The corresponding author can be contacted via email to request and receive the data of the current article, provided a valid and logical reason is stated.

## References

[vms370288-bib-0002] Becker, B. , S. Jordan , and W. Holzapfel . 2011. “Immunocompetence Profile of Aseel Breed of Chicken.” Indian Veterinary Journal 88: 23–25.

[vms370288-bib-0003] Cheema, M. , M. Qureshi , and G. Havenstein . 2003. “A Comparison of the Immune Response of a 2001 Commercial Broiler With a 1957 Randombred Broiler Strain When Fed Representative 1957 and 2001 Broiler Diets.” Poultry Science 82: 1519–1529. 10.1093/ps/82.10.1519 14601727

[vms370288-bib-0004] Cruse, J. M. , and R. E. Lewis . 2010. Atlas of Immunology. CRC Press.

[vms370288-bib-0005] Davison, F. , K. E. Magor , B. Kaspers , D. Fred , and A. Karel . 2008. “Structure and Evolution of Avian Immunoglobulins.” Avian Immunology 1: 107–127.

[vms370288-bib-0006] Davoodi, P. , A. Ehsani , R. Vaez Torshizi , and A. A. Masoudi . 2022. “New Insights Into Genetics Underlying of Plumage Color.” Animal Genetics 53, no. 1: 80–93. 34855995 10.1111/age.13156

[vms370288-bib-0007] Dawson, L. C. , T. M. Widowski , Z. Liu , A. M. Edwards , and S. Torrey . 2021. “In Pursuit of a Better Broiler: A Comparison of the Inactivity, Behavior, and Enrichment Use of Fast‐and Slower Growing Broiler Chickens.” Poultry Science 100, no. 12: 101451. 10.1016/j.psj.2021.101451 PMC8507195 34634710

[vms370288-bib-0008] de Klerk, B. , M. Emam , K. A. Thompson‐Crispi , M. Sargolzaei , J. J. van der Poel , and B. A. Mallard . 2018. “A Genome‐Wide Association Study for Natural Antibodies Measured in Blood of Canadian Holstein Cows.” BMC Genomics [Electronic Resource] 19: 694. 30241501 10.1186/s12864-018-5062-6 PMC6150957

[vms370288-bib-0009] Divya, D. , S. T. Viroji , M. Rao , P. Gnana , and J. Narasimha . 2018. “Immunocompetency of Two Chicken Backyard Variety With 25% Aseel Inheritance.” ThePharma Innovation journal 7, no. 2: 242–245.

[vms370288-bib-0010] Emam, M. , H. Mehrabani‐Yeganeh , N. Barjesteh , et al. 2013. “The Influence of Genetic Background Versus Commercial Breeding Programs on Chicken Immunocompetence.” Poultry Science 93: 77–84. 10.3382/ps.2013-03475 24570426

[vms370288-bib-0011] Fitzgerald, S. , S. Vale , and A. Maclean‐Tooke . 2019. “Measurement of the IgM and IgG Autoantibody Immune Responses in Human Serum Has High Predictive Value for the Presence of Colorectal Cancer.” Gastroenterology 156, no. 4: 123–135. 10.1016/j.clcc.2018.09.009 30366678

[vms370288-bib-0012] Gong, L. , B. Wang , X. Mei , et al. 2018. “Effects of Three Probiotic Bacillus on Growth Performance, Digestive Enzyme Activities, Antioxidative Capacity, Serum Immunity, and Biochemical Parameters in Broilers.” Animal Science Journal 89, no. 11: 1561–1571. 30198073 10.1111/asj.13089

[vms370288-bib-0013] Gross, W. , P. Siegel , R. Hall , C. Domermuth , and R. DuBoise . 1980. “Production and Persistence of Antibodies in Chickens to Sheep Erythrocytes. 2. Resistance to Infectious Diseases.” Poultry Science 59: 205–210. 10.3382/ps.0590205 6997852

[vms370288-bib-0014] He, J. , H. Pan , W. Liang , et al. 2017. “Prognostic Effect of Albumin‐to‐Globulin Ratio in Patients With Solid Tumors: A Systematic Review and Meta‐Analysis.” Journal of Cancer 8, no. 19: 4002–4010. 29187875 10.7150/jca.21141 PMC5706002

[vms370288-bib-0015] Heaton, S. M. , N. A. Borg , and V. M. Dixit . 2016. “Ubiquitin in the Activation and Attenuation of Innate Antiviral Immunity.” Journal of Experimental Medicine 213: 1–13. 26712804 10.1084/jem.20151531 PMC4710203

[vms370288-bib-0016] Hinkle, J. , and K. Cheever . 2014. Brunner & Suddarth's Handbook of Laboratory and Diagnostic Tests. 2nd ed. Wolters Kluwer Health, Lippincott Williams & Wilkins.

[vms370288-bib-0017] Hofmann, T. , S. S. Schmucker , W. Bessei , M. Grashorn , and V. Stefanski . 2020. “Impact of Housing Environment on the Immune System in Chickens: A Review.” Animals 10, no. 7: 1138. 32635616 10.3390/ani10071138 PMC7401558

[vms370288-bib-0018] Ismoyowati, I. , A. Darmanto , E. Tugiyanti , et al. 2022. “The Effects of Native Chicken Strains and Feed Addives on Immunity, Kidney Functions, and Blood Protein.” Journal of the Indonesian Tropical Animal Agriculture 47, no. 4: 277–289.

[vms370288-bib-0019] Kaewsatuan, P. , C. Poompramun , S. Kubota , et al. 2022. “Comparative Proteomics Revealed Duodenal Metabolic Function Associated With Feed Efficiency in Slow‐Growing Chicken.” Poultry Science 101, no. 6: 101824. 10.1016/j.psj.2022.101824 PMC8987610 35395531

[vms370288-bib-0020] Kashap, A. , R. A. S. Dalvi , and P. M. Kapale . 2017. “Study of Serum Biochemical Metabolites During Late Laying Phase of Layer Chicken.” Indian Research Journal of Extension Education 17: 5–9.

[vms370288-bib-0021] Leandro, N. , R. Ali , M. Koci , et al. 2011. “Maternal Antibody Transfer to Broiler Progeny Varies Among Strains and Is Affected by Grain Source and Cage Density.” Poultry Science 90: 2730–2739. 10.3382/ps.2011-01573 22080011

[vms370288-bib-0022] Li, Z. , K. E. Nestor , Y. M. Saif , and J. W. Anderson . 2000. “Antibody Responses to Sheep Red Blood Cell and *Brucella abortus* Antigens in a Turkey Line Selected for Increased Body Weight and Its Randombred Control.” Poultry Science 79: 804–809. 10.1093/ps/79.6.804 10875759

[vms370288-bib-0023] Loh, R. K. , S. Vale , and A. Maclean‐Tooke . 2013. “Quantitative Serum Immunoglobulin Tests.” Australian Family Physician 42, no. 4: 195–198. 23550242

[vms370288-bib-0025] Martin, L. B. , P. Han , J. Lewittes , J. R. Kuhlman , K. C. Klasing , and M. Wikelski . 2006. “Phytohemagglutinin‐Induced Skin Swelling in Birds: Histological Support for a Classic Immunoecological Technique.” Functional Ecology 20: 290–299.

[vms370288-bib-0026] Mengesha, M. 2012. “Indigenous Chicken Ecotypes: Genetic Potentials, Opportunities and Challenges for Ethiopian Farmers.” Asian Journal of Poultry Science 6, no. 2: 56–64.

[vms370288-bib-0027] Mohammadi‐Tighsiah, A. , A. Maghsoudi , F. Bagherzadeh‐Kasmani , M. Rokouei , and H. Faraji‐Arough . 2018. “Bayesian Analysis of Genetic Parameters for Early Growth Traits and Humoral Immune Responses in Japanese Quail.” Livestock Science 216: 197–202.

[vms370288-bib-0028] Morammazi, S. , and H. Habibi . 2017. “Sequence Variation in GAL1 and GAL2 Genes in Khuzestan Local Chickens.” European Online Journal of Natural and Social Sciences 6: 508–515.

[vms370288-bib-0029] Olaniyi, O. A. , O. A. Oyenaiya , O. M. Sogunle , O. S. Akinola , O. A. Adeyemi , and A. O. Ladokun . 2012. “Free Range and Deep Litter Housing Systems: Effect on Performance and Blood Profile of Two Strains of Cockerel Chickens.” Tropical and Subtropical Agroecosystems 15, no. 3: 511–523.

[vms370288-bib-0030] Pathak, P. , V. K. Nayak , and B. A. Ganaie . 2018. “Comparison of Various Immune Responsiveness Traits in Divergent Stocks of Chicken: A Review.” Journal of Pharmacognosy and Phytochemistry 7: 1045–1050.

[vms370288-bib-0031] Pinard, M. H. , J. Van Arendonk , M. Nieuwland , and A. Van der Zijpp . 1992. “Divergent Selection for Immune Responsiveness in Chickens: Estimation of Realized Heritability With an Animal Model.” Journal of Animal Science 70: 2986–2993. 1429274 10.2527/1992.70102986x

[vms370288-bib-0032] Qureshi, M. , and G. Havenstein . 1994. “A Comparison of the Immune Performance of a 1991 Commercial Broiler With a 1957 Randombred Strain When Fed “Typical” 1957 and 1991 Broiler Diets.” Poultry Science 73: 1805–1812. 10.3382/ps.0731805 7877936

[vms370288-bib-0033] Radhika, R. , D. Thyagarajan , P. Veeramani , and S. Karthickeyan . 2017. “Aseel, Kadaknath and White Leghorn Chicken Immune Response to Variation in Sheep Red Blood Cell.” Indian Journal of Pure & Applied Biosciences 5: 335–340.

[vms370288-bib-0034] Raeesi, V. , A. Ehsani , and R. V. Torshizi . 2019. “Genomic Loci Associated With Antibody‐Mediated Immune Responses in an F2 Chicken Population.” Animal 13: 1341–1349. 30451125 10.1017/S1751731118003014

[vms370288-bib-0036] Rauw, W. M. 2012. “Immune Response From a Resource Allocation Perspective.” Frontiers in Genetics 3: 267–267. 23413205 10.3389/fgene.2012.00267 PMC3571735

[vms370288-bib-0037] Sadr, A. , H. Habibi , S. Morammazi , and N. Eila . 2023. “Comparative Analysis of Immune Responses in Indigenous and Commercial Chickens.” Journal of Poultry Science 45, no. 2: 123–135.

[vms370288-bib-0038] Sahin, H. A. , and E. Ozturk . 2018. “Effects of Raw Propolis or Water and Ethanol Extracts of Propolis on Performance, Immune System, and Some Blood Parameters of Broiler Breeders.” Revista Brasileira De Zootecnia 47: e20170161. 10.1590/rbz4720170161.

[vms370288-bib-0039] Shahbazi, S. , S. Z. Mirhosseini , and M. N. Romanov . 2007. “Genetic Diversity in Five Iranian Native Chicken Populations Estimated by Microsatellite Markers.” Biochemical Genetics 45: 63–75. 17203406 10.1007/s10528-006-9058-6

[vms370288-bib-0040] Sørensen, P. 2010. “Chicken Genetic Resources Used in Smallholder Production Systems and Opportunities for Their Development.” Food and Agriculture Organization of the United Nations. FAO Smallholder Poultry Production Paper, No. 5.

[vms370288-bib-0041] Suganthi, R. U. 2014. “Immunocompetence of Indigenous Chicken: A Review.” International Journal of Science, Environment and Technology 3, no. 1: 1–10.

[vms370288-bib-0042] Tóthová, C. , E. Sesztáková , B. Bielik , and O. Nagy . 2019. “Changes of Total Protein and Protein Fractions in Broiler Chickens During the Fattening Period.” Veterinary World 12, no. 4: 598–604. 31190717 10.14202/vetworld.2019.598-604 PMC6515826

[vms370288-bib-0044] Vaillant, J. A. A. , Z. Jamal , and K. Ramphul . 2022. “Immunoglobulin.” In StatPearls. StatPearls Publishing. 30035936

[vms370288-bib-0045] Vali, N. 2008. “Indigenous Chicken Production in Iran: A Review.” Pakistan Journal of Biological Sciences 11: 2525–2531. 19260329 10.3923/pjbs.2008.2525.2531

[vms370288-bib-0046] Van der Zijpp, A. , and F. Leenstra . 1980. “Genetic Analysis of the Humoral Immune Response of White Leghorn Chicks.” Poultry Science 59: 1363–1369. 10.3382/ps.0591363 7393851

[vms370288-bib-0047] Wegmann, T. G. , and O. Smithies . 1966. “A Simple Hemagglutination System Requiring Small Amounts of Red Cells and Antibodies.” Transfusion 6: 67–73.

[vms370288-bib-0048] Yadav, S. P. , T. Kannaki , R. Mahapatra , et al. 2018. “In Vivo Cell‐Mediated Immune, Hemagglutination Inhibition Response, Hematological and Biochemical Values in Native vs. Exotic Chicken Breeds.” Poultry Science 97: 3063–3071. 10.3382/ps/pey182 29889283

[vms370288-bib-0049] Ye, Y. , W. Chen , M. Gu , et al. 2020. “Serum Globulin and Albumin to Globulin Ratio as Potential Diagnostic Biomarkers for Periprosthetic Joint Infection: A Retrospective Review.” Journal of Orthopaedic Surgery and Research 15, no. 1: 1–7. 33028348 10.1186/s13018-020-01959-1 PMC7539494

